# *Nurturing Creativity whilst Caring*: Participatory Action Research with family carers and a Recovery College

**DOI:** 10.3389/fpsyt.2025.1607560

**Published:** 2025-08-15

**Authors:** Bryher Bowness, Samina Begum, Sarah Bicknell, Lana Samuels, Sudhir Shah, Stephanie Hess, Karen Martin, Debbie Bark, Claire Henderson, Vanessa Lawrence

**Affiliations:** ^1^ Department of Health Service & Population Research, Institute of Psychiatry, Psychology & Neuroscience, King’s College, London, United Kingdom; ^2^ SLaM Recovery College, South London and Maudsley National Health Service (NHS) Foundation Trust, London, United Kingdom; ^3^ Independent Researcher, Bradford, United Kingdom; ^4^ Research and Development, Birmingham & Solihull Mental Health National Health Service (NHS) Foundation Trust, Birmingham, United Kingdom; ^5^ Independent Researcher, London, United Kingdom; ^6^ Oxfordshire Recovery College, Restore, Oxfordshire, United Kingdom

**Keywords:** Recovery College, family carer, Participatory Action Research, coproduction, creativity, self-care, mental health, relational recovery

## Abstract

**Introduction:**

The benefits of attending Recovery Colleges for mental and social wellbeing are well-documented, but the experiences of family carers (roughly 6–11% of students) are underexplored. Family carers report that attending courses supports their own wellbeing and recovery journeys, but also call for greater recognition and relevant provision from Recovery Colleges.

**Materials and methods:**

This Participatory Action Research project was codesigned by a Family Carers Advisory Group, an academic researcher, and staff at a Recovery College in England. We aimed to expand Recovery College provision to promote family carers’ wellbeing, by coproducing and coevaluating a creative course exploring self-care. We collected data through online feedback forms, fieldnotes, photographs and participatory reflective sessions, and collaboratively explored the family carers’ experiences of the course through inductive reflexive thematic analysis.

**Results:**

Seven family carers enrolled on our three-part online course *Nurturing Creativity Whilst Caring*. They shared photographs of the creative and self-care activities they took part in during the course, and gave feedback about what they found helpful and what could improve. We developed three themes summarizing their learning: ‘self-care as a family carer is complex, but there are small steps we can take to create time to nurture ourselves’, ‘creativity connects family carers with others and ourselves’, and ‘nurturing a creative mindset for caring’.

**Discussion:**

This example shows how Participatory Action Research can be an effective approach to designing courses for family carers in a Recovery College. We explore deeper understandings of self-care whilst caring, and the ways creativity can enable this and have wider reaching benefits. Our findings also add to the literature on implementing adult education in practice in Recovery College settings. Finally, we provide some implications for improving courses for family carers and future research.

## Introduction

Roughly 8.8 million people in the UK provide substantial informal support to family or friends with disability or ill-health, with around 13% of these caring for someone with a mental health difficulty (1). We use the term ‘family carer’, but recognize many people in this diverse group may not identify as such (2), or with any label, and often hold multiple roles. With the increasing pressure on formal healthcare provision in England, family carers are relied upon more to support those with mental ill-health (3, 4). There is much research into the challenges of being a family carer (5). For example, caring can bring a range of distressing emotions, such as anger, anxiety, worry, and grief (6). A multitude of factors – e.g. stigma, time, financial pressures – may prevent accessing leisure activities and contribute to their social isolation (7–9). Subsequently, mental health carers are more likely to experience poor emotional and physical well-being (5, 10). This is compounded by a lack of recognition (11), information, and funding for support from healthcare services (7, 12), despite policy initiatives aiming to improve the support and information provided to family carers (13).

Family psychoeducation groups, providing information about mental illness, training in communication, and problem-solving, are perhaps the most evidenced intervention. These demonstrate significant benefits for caregiving, such as increased understanding and improved coping mechanisms, whilst reducing relapse rates (14–16). However, studies have found that fewer than half of family carers receive psychoeducation (17), with low uptake due to various barriers to access, such as referral processes and time-intensity (18). Delivering programmes in an online format is proposed as an option which may increase feasibility for family carers, although results thus far are inconclusive (19, 20). Also, most research has focused on how family psychoeducation can equip people for their caring role, with less emphasis on understanding their own wellbeing (21).

Another reason some family carers delay seeking support is feeling that taking time out or asking for help is a sign of failure or weakness, beliefs perhaps originating from internalized stigma of societal gender roles (9, 22). Given the time demands of caring, often on top of other commitments (23), family carers often struggle balancing their own needs with those of their relative and believe they must put the other person first (24). Promoting “self-care worthiness”, i.e. seeing yourself as deserving of nourishment (25), may be required for family carers to prioritize and make time for their own support (26). It is therefore vital to take an approach that emphasizes the inherent value of the family carers’ wellbeing, not solely helping them to sustain their caring role (18).

Therapeutic interventions, such as mindful self-compassion courses (27), mutual support groups (28) and collective narrative-based art therapy (29), show promise in helping family carers to process their distress. However, more may be needed to support their “growth beyond the catastrophic effects of mental illness” (30, p.527). Increasingly, mental health recovery, is understood as relational (31), whereby family carers also have their own recovery journeys, which are separate from and interlinked with the people they support (32, 33). Elements described as important in family carers’ recovery include rediscovering an identity besides caring (11), rebuilding a positive sense of self, and meeting others with shared concerns and experiences (34). But one large study of family carers recently found that the majority scored low on a measure of personal recovery, and felt they were not empowered to recover on their own (35). This calls for more research into how we can promote personal recovery for family carers.

Recovery Colleges have the potential to equip family carers for their caring, whilst simultaneously supporting their wellbeing needs (36). Taking an adult educational approach, Recovery Colleges are international innovations providing recovery-oriented courses. There are over 88 Recovery Colleges in England, used by over 360,000 students (37). Distinguished from psychoeducation by their pedagogical underpinnings (38), such as transformative learning theory techniques (39), and in their core principles of coproduction (40). Students may be mental health service users (a majority), staff, family carers, and community members (and often identify with a combination of these roles), and usually they all learn together. The psychological and social benefits of attending Recovery Colleges for students as a whole are well-documented [e.g., (41, 42)]. Some of these transformative effects may be applicable for the 6-11% of students (43) who identify as family carers, but more research is needed (41).

A recent focus group study of 23 family carers from Recovery Colleges across found they too reported increased hope, opportunities and empowerment (44). Participants of this study appreciated the unique Recovery College approach, particularly the educational focus and learning from lived experience. Many family carers initially attended courses specifically about caring (available in roughly 70% of Recovery Colleges in England); these built their skills for caring but also helped them realize the importance of taking care of themselves too, which led to them pursuing other courses on the curriculum. However, family carers often felt overlooked by Recovery Colleges, calling for more courses tailored to their learning and accessibility needs. We found one other single-site case study evaluating the development and delivery of a family carer-specific online course (45). Their course focused on promoting an understanding of personal recovery and how this can be built into the caring role, but found a strong theme in participants’ experiences was their recognition of the importance of attending to their own needs too. Designing and delivering courses that consider how we promote family carer recovery journeys, as well as those of service users, would help Recovery Colleges attain their foundational aspirations of inclusivity (46).

### Research aims

Our overall aim was to gain a deeper understanding of the ways Recovery Colleges can benefit mental health family carers, and how to implement these. Building on existing research, the specific aims of this research project were to;

a. Identify one or more ways to promote recovery for family carers, and
b. Co-produce and co-evaluate this approach within a Recovery College setting

## Methods and materials

### Participatory Action Research

**Figure 1:**
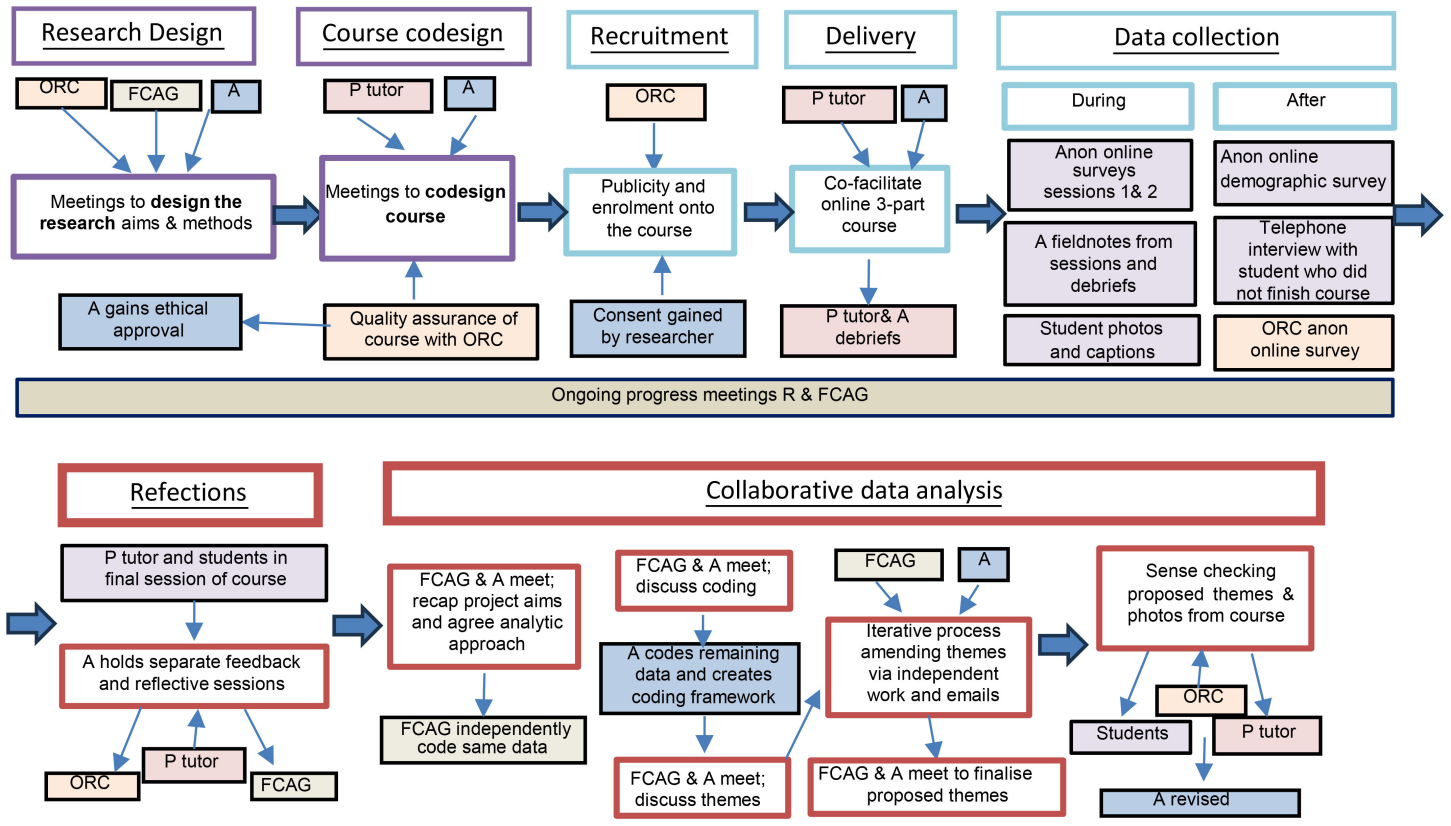
Stages of involvement throughout the PAR project.

Involving family carers in the design and delivery of their provision is essential to enhancing effectiveness and acceptability (47), whilst also furthering their empowerment through the process (48, 49). This led to using a Participatory Action Research design to address the project aims (a further exploration of these collaborative methods will be reported elsewhere). Participatory Action Research (PAR) originated in contrast to traditional research approaches, as an emancipatory research paradigm, aiming to empower those who may previously have been excluded. Academic researchers and community members with lived experience form collaborative relationships through which they co-construct contextual knowledge for action (50, 51). Together, they share in the decision-making throughout the research process, which consists of cycles of *identifying aims*, *planning*, *acting*, and *reflecting* to achieve change (52). **Figure 1** provides an overview of each stage in the project and who was involved.

#### Research team

The academic researcher was a part-time PhD student with a background in mental health nursing, employment in a Recovery College, and her own lived experience of mental ill-health. She recruited four coresearchers to a Family Carers Advisory Group from different cultural backgrounds who brought diverse experiences of caring and living with familial mental illness. Two had been on the academic researcher’s previous project, and they all had varying experiences with research, attending or using Recovery Colleges. The academic researcher then approached Oxfordshire Recovery College (ORC), building on her existing relationship as one of their students. ORC had previously collaborated in university research projects and had recently developed an innovative arts-based project, their Library of Life (53). The manager and tutor coordinator joined the group planning sessions. They then enlisted one of their experienced volunteer peer tutors who had prior caring responsibilities, her own mental health journey, and a background in education, to codesign and cofacilitate the course with the academic researcher.

#### Setting

ORC is a charity-funded, community-oriented Recovery College (37), providing free, online and classroom courses, to anyone aged 18 years and over living in Oxfordshire. Established in 2015, ORC has around 450 active students, of whom roughly 8% have enrolled primarily as family/carer/supporter. Since 2021, ORC have collaborated with the local Carers Support Service at Rethink Mental Illness to deliver a 1.5-hour online course ‘Introduction to the caring role’, open to all their students.

### Identifying aims – an approach to promote recovery for family carers

To address the first of our research aims (identify an approach to promote family carer recovery), we held five online meetings between the academic researcher, the Family Carers Advisory Group, and members of ORC. During these meetings, we discussed what PAR is, the previous research about family carers and Recovery Colleges (44), what the family carer coresearchers felt would help others with caring responsibilities, and how this might fit the context of ORC. We invited representatives from the Carers Support Service, who described the current provision and unmet needs reported by local family carers (recommended in suggestions for how Recovery Colleges can improve their provision for this group (44). A summary of each meeting was circulated afterwards for comments from the group, which helped the group reach a sense of shared understanding. Where there were different opinions regarding decisions in meetings, the researcher helped to find commonality between these views in follow-up emails, which aided the group to create an option they all agreed upon.

Through these planning sessions, we decided to address the research aims by coproducing a new Recovery College course for family carers, exploring self-care through creative methods.

#### Rationale behind the course

In the absence of published guidelines for tailoring Recovery College courses to family carers, our rationale for the course format and general objectives was strongly influenced by the recommendations from previous research (44), the lived experience and practice experience of the research team.

The family carer coresearchers suggested we focus on self-care, a need also highlighted by the local Carers Support Service. Indeed, other Recovery Colleges deliver courses such as ‘Caring for Carers’ (54). There is growing evidence for the role of developing self-care strategies in family carers’ recovery; becoming ‘self-care virtuosos’ is one way that has helped mental health family carers to ‘journey on’ (55), nurture hope (56), and help them to ‘feel human again’ (24). The family carer coresearchers proposed using creative methods in the course, as a form of self-care that was accessible, with the appeal of making tangible end products (57). Also, nurturing creative thinking and participating in creative activities is shown to enhance mental health by promoting numerous psychosocial benefits; emotion regulation, cognitive flexibility and social connectedness (58). ORC also reported that their creative courses were always popular with all their students, and felt this may perhaps be because there was less stigma attached to them. Family carer coresearchers suggested the students may be better able to express themselves through creative methods, whilst simultaneously generating insight into their experiences useful for the research. Indeed, creative methods have been used to give voice to family carers in previous PAR (59).

Finally, we agreed the course should be delivered online and be limited to three, short sessions, as this might be easier for family carers to attend amongst their other commitments (36, 60). Prior research suggests that students may find it harder to form social connections in digital courses (61). To maximize the likelihood of sharing experiences and the development of peer support (62), we limited the course to those who identified as family carers (current or past), unlike the typical mixed learning environment of all previous ORC courses.

### Planning – coproducing and coevaluating our approach in a Recovery College

#### Course codesign

We (the peer tutor and academic researcher) took a fluid and informal approach to codesign, building trust and understanding of each other through communication and respecting each other’s unique needs and strengths. We met every couple of weeks online over eight months, alongside using an online slide-sharing platform and emails. As part of their quality review process, we also met with the lead tutor and manager of ORC, who recommended minor adjustments to ensure all the activities were inclusive and would fit within the time.

Based in the initial course aims, we created learning objectives (**Table 1**), to structure our lesson plans and course materials.

**Table 1:** Course aims and learning objectives.

Aims for the course (codesigned by ORC and the Family Carers’ Advisory Group) • Increase awareness of the importance of self-care whilst caring and explore some of the barriers to this • Identify ways to fit creativity and self-care into their lives • Explore their caring role and their identity • Share ideas and connect with others with similar experiences Learning Objectives for *Nurturing Creativity whilst Caring* (codesigned by the course tutors) Session 1 *The importance of nurturing self-care* – students will… • Explore what self-care means to them • Learn about the importance of self-care whilst caring for others • Share barriers to self-care • Discuss ways to implement self-care in their own lives Session 2 *The importance of nurturing creativity* • Learn about the different kinds of creativity, and their benefits to mental wellbeing • Discuss individual ways they can be creative in their daily lives • Engage in a shared mindful creative activity and reflect on this Session 3 Our community of creative self-care • Reflect together on the take-home task and their experiences of practicing self-care and creativity during the course • Learn about self-care as a collective concept • Engage in a creative activity to explore how we can support each other • Reflect on the course and provide feedback for future iterations

*Specific course materials and lesson plans for how these learning objectives were achieved are available on request of the lead author.

#### The course: *Nurturing Creativity whilst Caring*

The end-product was *Nurturing Creativity whilst Caring*. The course covered content that combined current research about family carers’ recovery journeys and the benefits of creativity with stories and tips from the peer tutor’s lived experience. We intended to encourage everyday forms of creativity (such as admiring small details, having stimulating conversations, and trying new things) as these are thought to have more mental health benefits than cultivating artistic talents (63). We used transformational learning techniques [in accordance with the pedagogy of recovery education 38)], such as group discussions and experiential activities. For example, session two involved a mindful photography activity, for students to go away and pay attention to an object before sending a picture of this to the tutors for group discussion. This technique has been used effectively to encourage positive affect and help ‘seeing through new eyes’ (64), and we also hoped to signpost students to ORC’s more in-depth ‘Mindful Photography’ course. In and between sessions, students started creating an individual ‘Wellness A-Z’, a technique used by the peer tutor whilst she was caring. To facilitate this, a creative materials resource pack was posted to each student (i.e. small notebook, stickers, coloring pens).

#### Planning our evaluation

Following the codesign, the Family Carers’ Advisory Group and ORC met with the academic researcher to plan a protocol for a mixed-methods evaluation to assess whether the course had met our research aims i.e. to identify and coproduce a way Recovery Colleges can promote recovery for family carers. We brought complementary knowledge of feedback mechanisms that work in practice at ORC, research methods sensitive to the needs of family carers, and the academic researcher’s requirements for the project. We codesigned brief online feedback forms and questions for a group reflective discussion in the final session, as there are no agreed-upon measures for recovery in family carers (65). To minimize the burden to busy family carers wherever possible (66), we tried to capture reflections during the sessions and limit the use of questionnaires. We selected the least intrusive methods of data collection, i.e. choosing not to record sessions, to maintain the safe space of the course, thought to be a crucial mechanism of change in Recovery Colleges (42). Ethical approval for the study was obtained by the academic researcher from King’s College, London, Research Ethics Committee on 08/04/2024 (LRS/DP-23/24-40819), which also required ORC and the academic researcher to sign a university collaboration agreement.

### Acting

#### Recruitment

Participants, hereon referred to as students, were eligible if they enrolled with ORC (i.e. over 18 years old and living in Oxfordshire) and identified as supporting someone with mental ill-health. Filling the course was challenging due to the small pool of students at ORC who identify as family carers (roughly 8%). Common in PAR approaches, we utilized community-based recruitment methods with those already working with family carers (66). ORC publicized the course through their physical, online prospectus and mailing list, and mentioned it to students they thought might be interested. Details of the course were also advertised by the local Carers Support Service and the local mental health services’ Carers Network. A condensed taster session was delivered during Carers Week for further publicity.

After expressing an interest in the course and establishing their eligibility, potential students consented to ORC passing their contact details to the academic researcher. They were then offered an introductory phone call and sent an information sheet and an online consent form, before ORC enrolled them onto the course.

#### Data collection

After both session one and two, students were sent brief anonymous online feedback forms. These asked them to rate the session with one of three emojis, what they had learnt in the session, and anything they didn’t like or would change. After the whole course, the ORC sent their usual online anonymous feedback form, and the academic researcher sent an anonymous demographic questionnaire and longer end-of-course survey. This contained multiple-choice questions and open questions designed to assess student experience, effectiveness of course format, achievement of learning objectives, and whether the course had met initial aims (see **Table 1**).

Students who did not complete the final session were given a choice of how they would like to give feedback the following week – one chose the end-of-course survey, and the other chose an unstructured telephone interview where the academic researcher took notes. Because the online sessions were not recorded, the academic researcher took anonymous fieldnotes during the course. Anonymous student comments from the online chat were also saved.

Students emailed photographs of their ‘Wellness A-Z’ booklets, self-care activities and creations whilst caring. Any images that had identifiers of other people were deleted. Along with any accompanying explanations, photographs were pseudo-anonymized and saved on a password-protected shared drive. In the final session, a montage of these photos was shared for group reflection, where they discussed their meaning. A separate information sheet and consent form were completed after session two about using the images that students offered of their artwork during the course. One student asked the researcher not to share her images, whilst the other six consented to sharing with the group, the research team and in research publications (one wished to be named with her artwork). Each student was sent a booklet of a compilation of the photographs at the end of the course.

### Reflecting

A group reflective discussion was incorporated into the final session, where the academic researcher asked some questions (that had been preplanned with the Family Carers Advisory Group). These were open-ended, intended to gain a deeper understanding of the students’ experiences and also to elicit their own interpretations on whether the course met the course aims and objectives. Students were also prompted to discuss the meaning behind the photographs they had taken and anything they noticed or themes amongst these altogether. This informal interpretation acted as a participatory means of analyzing visual data of their experiences during the course, which fed into our formal analysis.

The tutors (i.e. the academic researcher and peer tutor) met to debrief after each session, during which the academic researcher took notes. We discussed how we felt the students experienced the session, what went well, and what we might improve, as well as facilitation dynamics and personal feelings. At the end of the course, we held a longer reflective session together, which the academic researcher recorded and transcribed to incorporate into the formal qualitative analysis.

The academic researcher also facilitated sessions with the ORC and then the Family Carers’ Advisory Group to reflect on the course feedback, taking notes on these meetings to gather initial interpretations before beginning thematic analysis.

#### Data analysis

**Figure 2:**
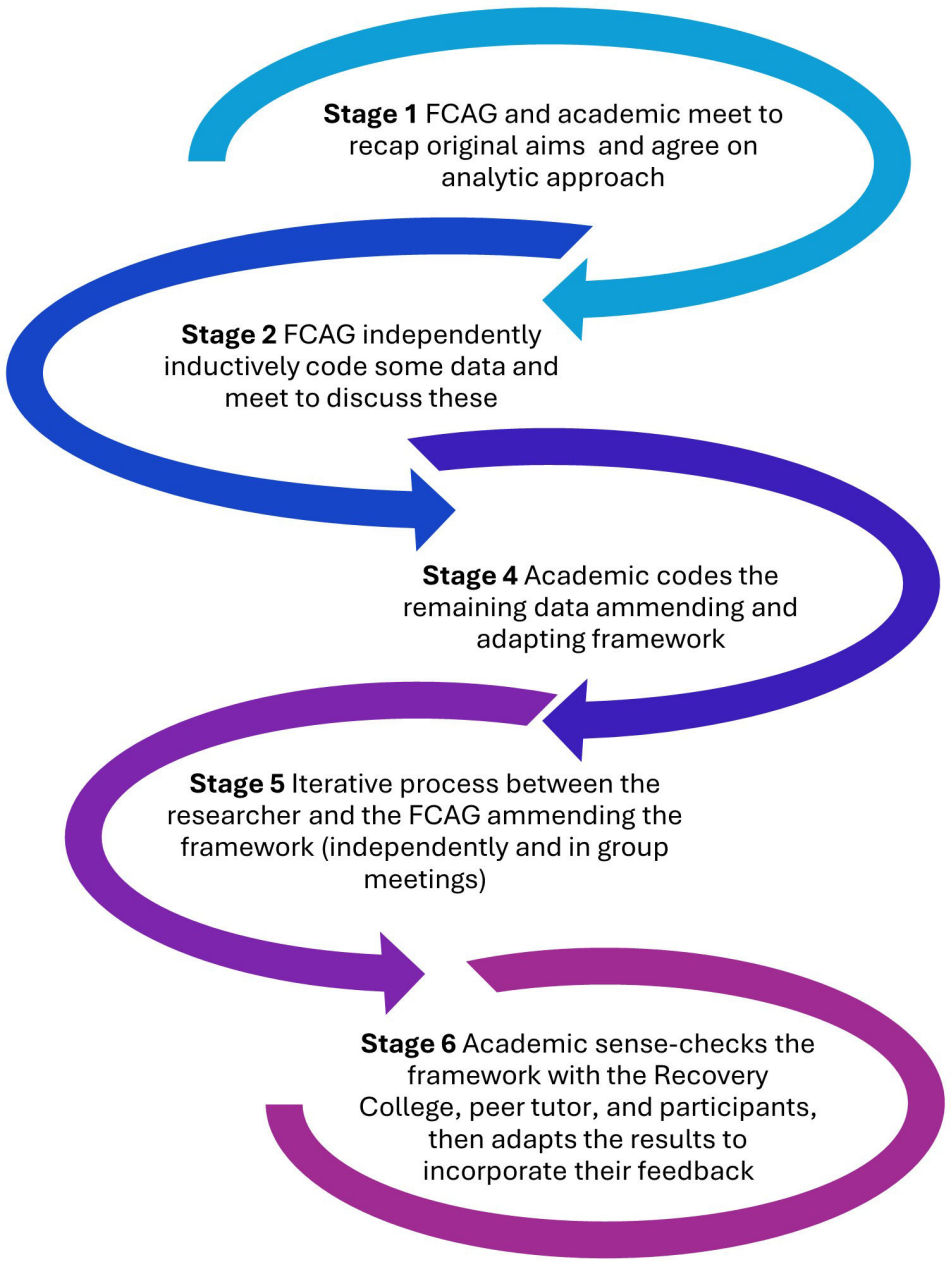
CDA process.

Collaborative Data Analysis (CDA) brings together multiple interpretations and enables critical reflection on assumptions to generate a shared understanding. CDA can involve community research partners to different extents (67). The peer tutor and ORC wished to be consulted in the later stages of finalizing themes and recommendations, whereas all members of the Family Carers’ Advisory Group wished to be involved with the development and application of the coding framework. We decided together on the practicalities for the analytic strategy, basing this largely on the CDA methods of the academic researchers’ previous study (44) and the coresearchers’ prior experiences of coding. **Figure 2** depicts each stage of the analysis process.

We used reflexive thematic analysis (68), relatively well-suited to participatory approaches (69) and applicable to data sets in multiple forms. The following sources of data were compiled together to include in the reflexive thematic analysis;

- Online anonymous surveys - feedback form session one, feedback form session two, ORC survey and end-of-course surveys
- Photographs submitted by the students between and after sessions, together with their captions
- Researcher fieldnotes from each session (including the group reflection in the final session)
- Researcher field notes from the telephone interview
- Verbatim text from the recording of the end-of-course tutor debrief
- Verbatim written text from the online chat in each of the sessions

To address our second research aim of evaluating whether our chosen approach (coproducing a Recovery College course focusing on creativity and self-care) was experienced as helpful for family carer recovery, we used inductive coding, driven by the data of the students’ own experiences. We then applied framework analysis (70) to help us look for patterns across the dataset simultaneously, whilst considering the aims for the research. We also sent students a copy of our preliminary thematic framework, accompanied by a printed booklet of their photos from the course, with the opportunity to comment (and an offer of a gift voucher reimbursement). One student responded, confirming that our interpretations resonated with her experiences. Each time the family carer coresearchers, ORC, or students provided comments on the codes or themes, the framework was revised to emphasize those points they felt resonated most with their experiences. Although the budget limited the extent to which the Family Carers’ Advisory Group could be involved in the write-up of the findings, all members commented on paper drafts as coauthors.

## Results

Enrolment in the course required multiple publicity and follow-up emails from the ORC team. Nine students expressed interest in the course, and seven enrolled (two could not make the dates due to other commitments). Six were already students at the College, whilst one person heard about the course through the Carers’ Support Service and enrolled especially. All the students were females supporting family members (demographics for the four students who completed the anonymous survey emailed after the course are shown in **Table 2**). One student could not attend the final session because of planned caring commitments, and another missed this due to health concerns. They were both sent the final session content then one student completed the end-of-course online survey and submitted photographs, the other gave a reflective interview by telephone.

**Table 2:** Demographics survey.

Question	Answer	*N=4*
What is your gender?	Female	4
What is your employment status?	Unemployed	2
	Retired	1
	Unable to work	1
How old are you?	31-45	2
	Over 60	2
How would you describe your ethnic origin?	White	4
Do you support someone who struggles with their mental health? If so, what is your relationship to that person?	Mother	1
	Mother	1
	Spouse	1
	Daughter, granddaughter, niece, sister	1
What is the nature of the difficulty that person struggles with?	Autism and anxiety	1
	Psychosis	1
	Bipolar	1
	Dyslexia, autism, multiple health conditions	1
How long have you been supporting that person?	Several years	1
	At least 5 years	1
	More than 10yrs	1
	30 years	1

Responses to anonymous online demographic survey (n=4).

### Multiple-choice questions from the end-of-course online survey

**Figure 3:**
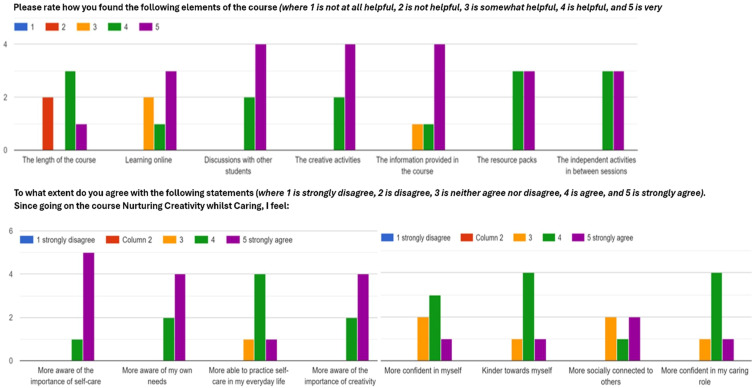
Bar chart showing students’ answers to the multiple-choice questions from the online end-of-course survey.

Six students completed the feedback form emailed after session one, and four completed this after session two and four completed the demographic survey (see **Table 3**). In total, six students completed the end-of-course feedback form. The majority of students (4/6) reported they found the information provided, the discussions, and the course material very helpful (see **Figure 3**). Feedback on the helpfulness of the online learning and length of the course was more mixed; two students found the length of the course was not helpful, and two found learning online was only somewhat helpful. In terms of knowledge and skills imparted from the course, most students strongly agreed that afterwards they felt more aware of the importance of self-care (5/6), the importance of creativity (4/6), and of their own needs (4/6). Most students agreed that the course helped them to be kinder to themselves (4/6) and feel more confident in their caring role (4/6). They were also in agreement that they felt more able to practice self-care in their everyday lives (4/6).

**Table 3:** Overview of themes and subthemes.

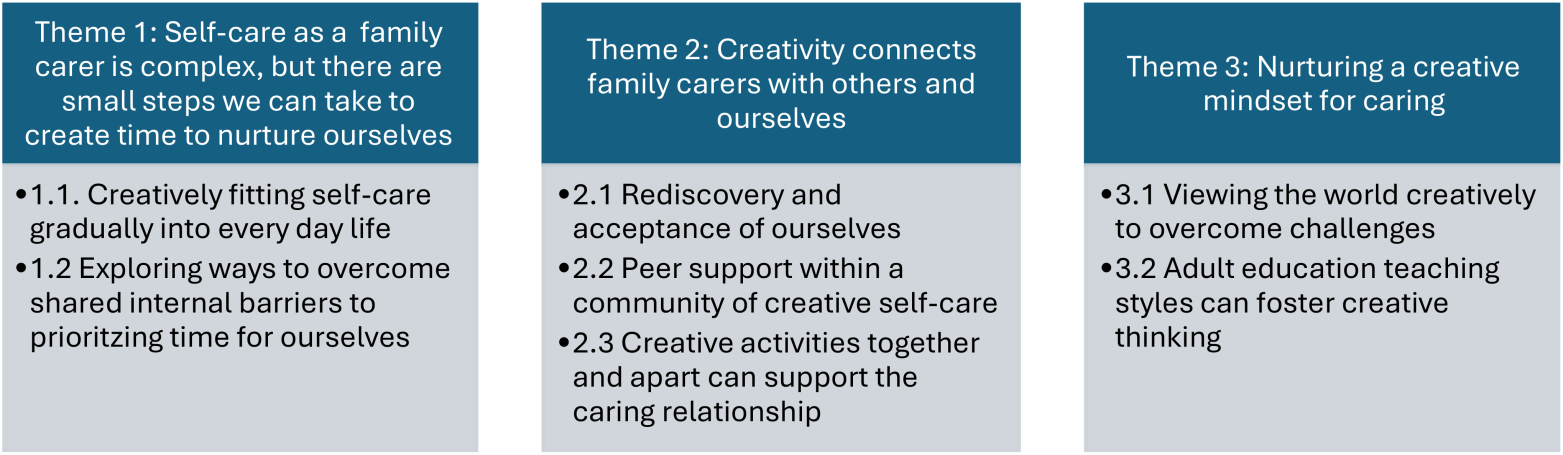

### Reflexive thematic analysis

We developed three themes and five subthemes relating to how our course helped support family carers’ wellbeing and recovery, drawn from the experiences and reflections of the course *Nurturing Creativity whilst Caring* (see **Table 4**).

**Table 4:** Coproduced recommendations for future creative courses for family carers.

• Sometimes people with caring responsibilities might have more challenges to participating in courses. Providing resources and activities to do at home may help them keep up the learning independently. • Online courses are more accessible for some family carers, and can still promote creativity and a sense of community. But for other students, virtual formats pose technical challenges and may be less engaging. People may require extra preparation and support from course tutors to join and benefit from the course. • Working in partnership with stakeholders and organizations that support family carers helps to ensure the course is relevant and reaches more family carers. It’s also essential to continue coproducing these with family carers. • These courses take a lot of time and resources, but the positive feedback we received indicates investment in nurturing creativity and self-care in family carers through Recovery Colleges is worth it

#### Theme 1: self-care as a family carer is complex, but there are small steps we can take to create time to nurture ourselves

##### Creatively fitting self-care gradually into everyday life

**Figure 4:**
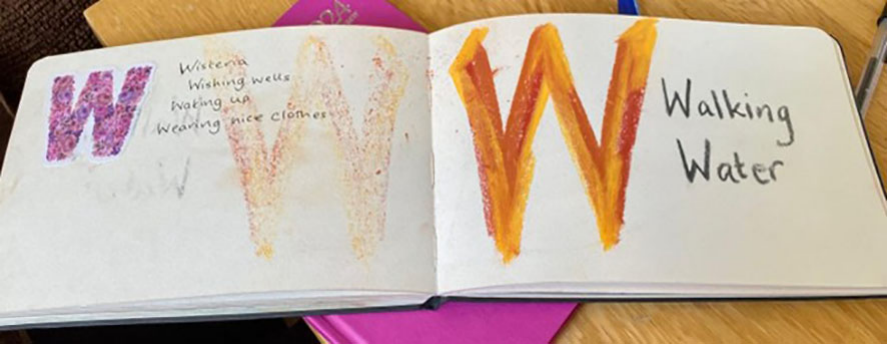
An example of a student’s ‘Wellness A-Z’ created during the course.

Students explored their personal definitions of self-care together through a group creative activity, which broadened and deepened understandings of the concept. A lot was involved for family carers to make time for themselves; *“activities for me, and all that is entailed to make this happen … self-care involves planning and ensuring there is a backup plan,”* (student, end-of-course feedback form 4). They found it useful sharing time management and scheduling strategies, and this stimulated ideas to help students figure out what works for them. One student realized that the pressure they put on themselves to engage in additional self-care activities, such as exercising or crafting, led to procrastination and self-criticism. The group concluded that self-care whilst caring starts with small steps and requires daily practice. For some of the family carers, this meant just getting the basics right or staying on top of things (like attending appointments or getting enough sleep). Others achieved this by appreciating the everyday experiences, as shown in the students’ A-Z (**Figure 4**). Approaching self-care creatively helped the family carers ensure that the time they were able to make for themselves was spent purposefully doing things that recharged them.

##### Exploring ways to overcome shared internal barriers to prioritizing time for ourselves

**Figure 5:**
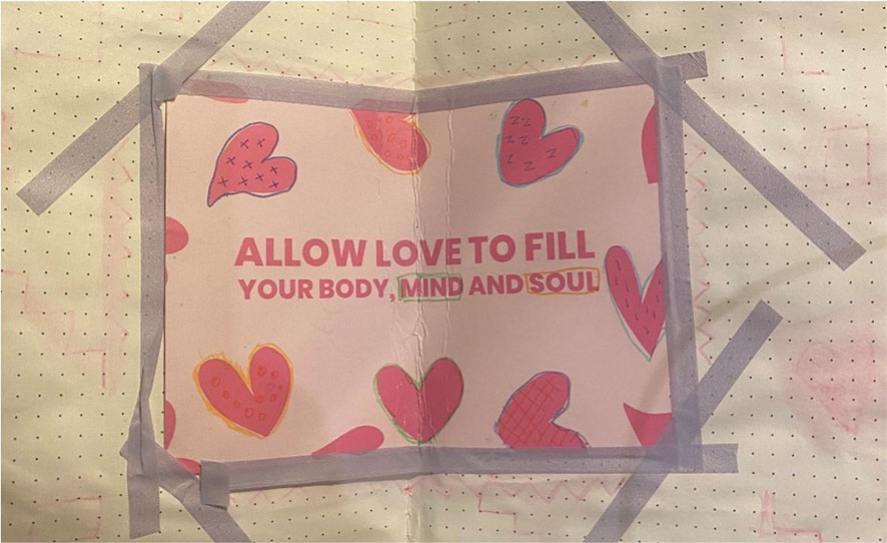
Student journal made during the course *“to encourage me through the present hard times of caring for a loved one”* (student caption in email accompanying the photo).

Family carers in our study reported feeling worry and guilt, as there often seemed to be a *“difficult choice between the needs of myself and the cared for”* (student, first session feedback form 4). The course gave ‘allowed’ them to take time for themselves. Students valued the mindfulness and creative activities as the opportunity to be still and a break from constantly doing. Despite their busy lives and responsibilities, some of the family carers even wished the course was longer. Following the course, students felt *“kinder to myself about needing a break”* (student, end-of-course feedback form 6). A take-home message was that family carers must prioritize self-care, but this requires committing to practicing self-compassion (see one student’s motivational journal in **Figure 5**).

#### Theme 2: creativity connects family carers with others and ourselves

##### Rediscovery and acceptance of ourselves

**Figure 6:**
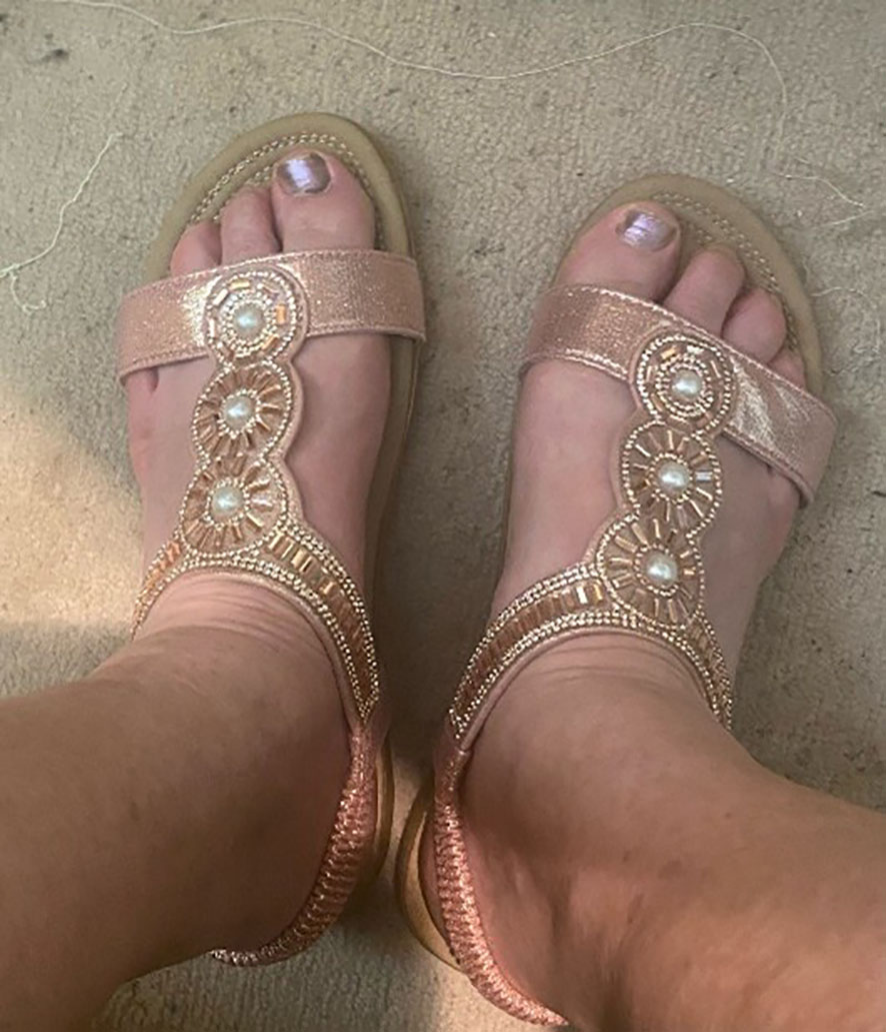
Student photograph ‘*my* sp*arkling shoes’* (student caption in email accompanying the photo).

The course gave students a space to spend time getting to know themselves, trying out activities because they enjoyed them, not because of all the ‘shoulds and musts’ they faced as family carers. Doing some of the creative things they used to do before they started caring helped them reconnect with their identity. One student liked creativity because it helped her to feel in control and achieve something, giving her confidence. Other students were more apprehensive before the course, because they believed they were not ‘creative’. However, exploring wider definitions of creativity as a way of thinking helped them view themselves in a new light; “*actually, when I think about it, I aced it”* (student quote, final session fieldnotes). They also found creative ways of expressing and celebrating themselves, such as spending time on their appearance (see **Figure 6**), thus practicing a broader definition of self-care as a *“radical acceptance of who I am”* (student quote, session two fieldnotes). By the end of the course, one student felt like *“sunshine trying to pop through the clouds”* (student quote from online chat, final session).

##### Peer support within a community of creative self-care

Much of the student feedback described the safe, unpressured and enjoyable environment of the courses, allowing them to try out new ideas. Together, they supported each other around some of the difficulties of caring and how to overcome barriers to both self-care and creativity; *“[This] gives me a sense of community that understands”* (student, end-of-course feedback form 2). Being with like-minded others normalized their worries and developed the family carers’ self-compassion. In the course, we also introduced a concept helpful in sustaining wellness called ‘the wisdom of we’, which prompted students to think about how others could inspire their creativity and support them to regularly practice self-care.

**Figure 7:**
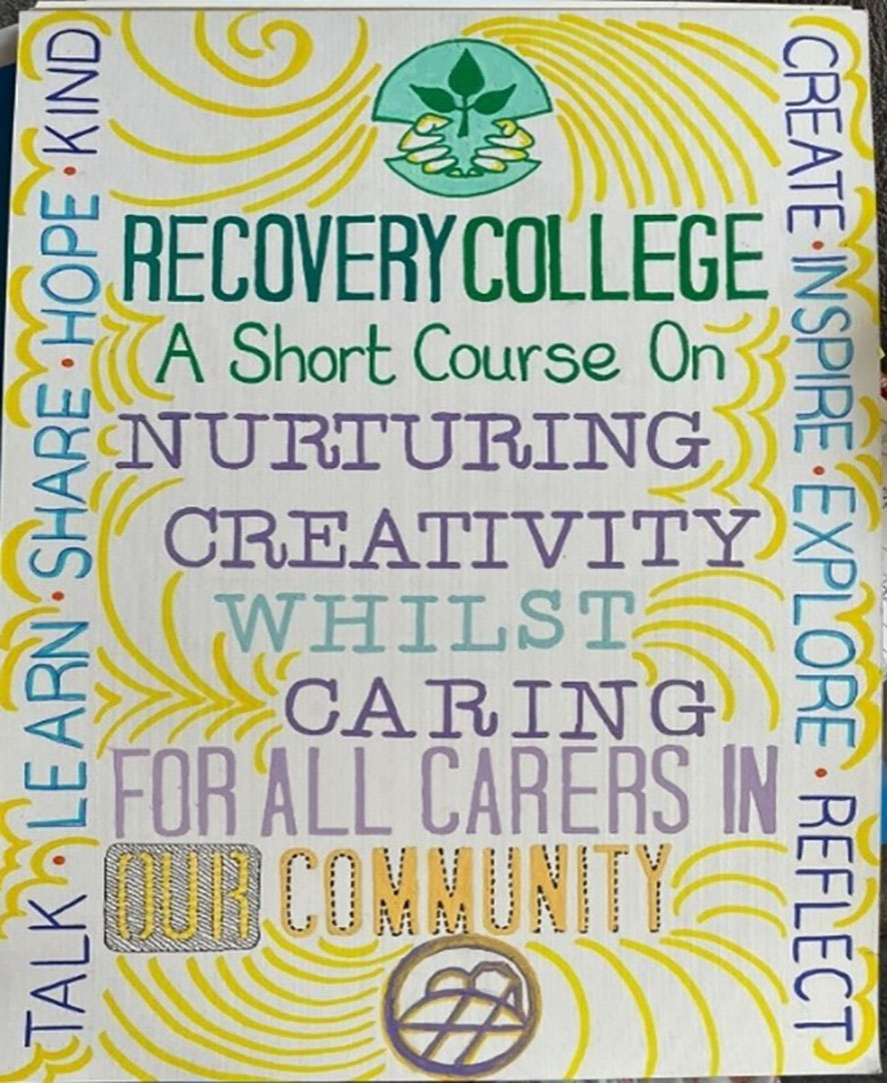
Louisa’s promotional poster.

For one student, it was her first time coming to Recovery College, and others said the course appealed because it was specifically for carers. After doing the course, they wanted to get more involved and had changed their perspective on what the wider Recovery College might have to offer them: *“Most courses at Recovery College I go along just as a carer to accompany my husband, but actually I can benefit from courses too.”* (student quote, final session fieldnotes). Another student was so passionate about finding ways for more family carers to benefit from the course that she designed the poster shown in **Figure 7**.

##### Creative activities together and apart can support the caring relationship

**Figure 8:**
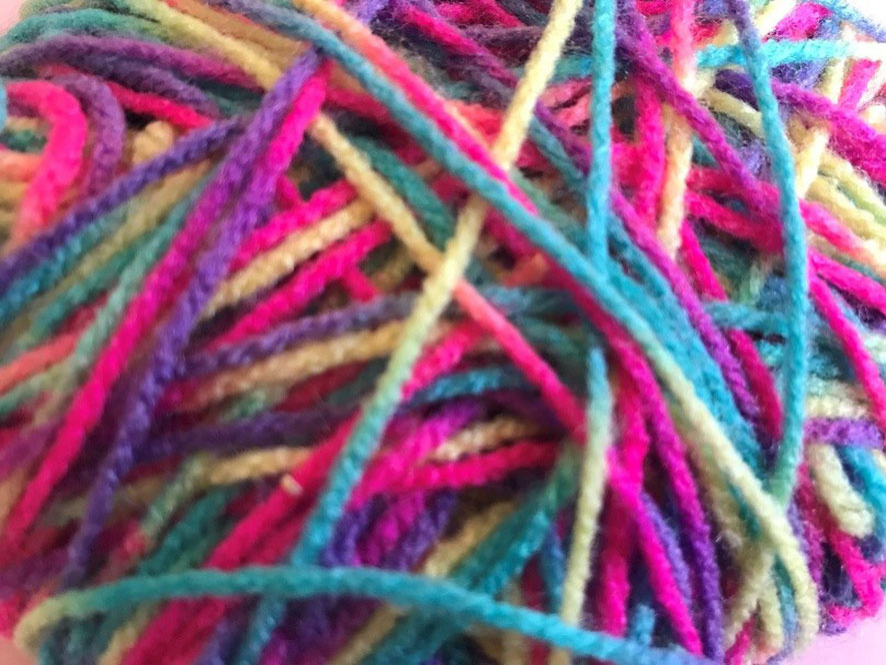
Student’s mindful photograph of wool as a self-care beginning with W.

Unanticipated by the tutors when they designed the course, many of the students reported trying the creative activities at home with the person they were caring for, which they both benefited from. Showing the art that she made during the course helped one student to communicate her own needs to her family. In contrast, family carers in our course also found that doing creative activities independently took their mind to a more peaceful place and helped them to *“look after myself by escaping the everyday challenges of life … more ‘craftive’ activities to balance my heavy caring responsibilities at present”* (student, end-of-course feedback form 5). Mindful creative activities encouraged students to be in the present moment and to feel less absorbed by caring. Reflecting on the role creativity can play in a family carer’s journey, one student described how crocheting during very difficult times had required her to slow down and ‘be’, rather than trying to do and fix things for the person they supported. Subsequently, she felt more able to process the complexity of mental ill-health, also helping her with caring. See **Figure 8**, a photograph of wool, which is also a metaphor for how the wellbeing of family carers and those they support are interwoven. 

#### Theme 3: nurturing a creative mindset for caring

##### Viewing the world creatively to overcome challenges

**Figure 9:**
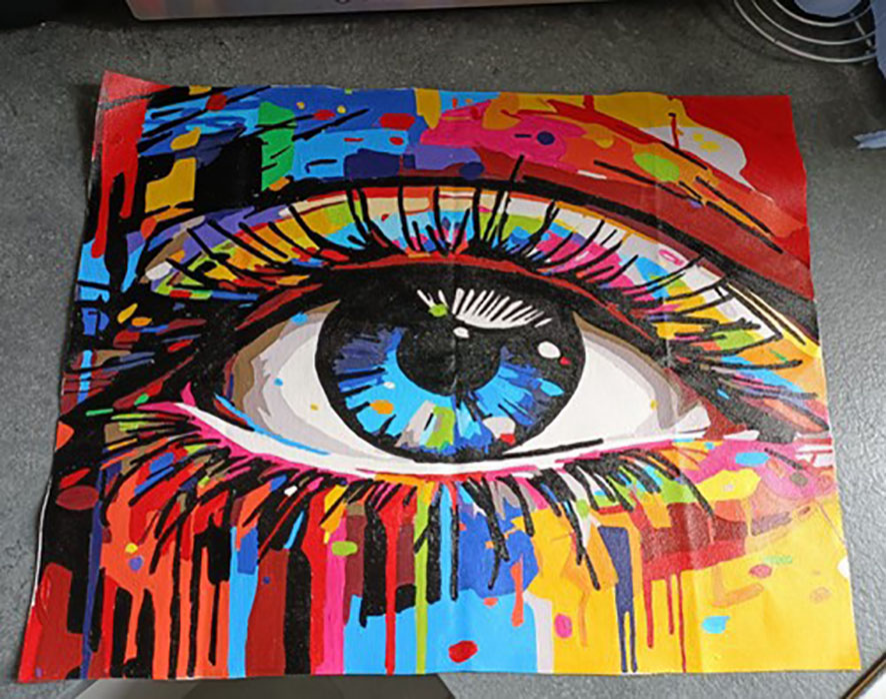
‘Watching’; painted by a student during the course to show self-care beginning with W.

In the second session, we thought about the multifaceted nature of creativity, introducing the idea that creativity can be a way of looking at things differently. Following the mindful photography activity, the students began to notice they were noticing new things, feeling inspired, being more curious and patient*, “looking at the world with eyes of wonder”* (student quote, final session fieldnotes). Many of them reflected on this “*shift in my mind”* (student, end-of-course feedback form 2) cultivated throughout the course. The image of a painting of an eye in **Figure 9** was shared by a student who found it difficult speaking in the group, but through creativity gained confidence to contribute and express themselves.

Persisting through the discomfort of some of the unfamiliar activities was “*a little challenging, which I think is good to help me think outside the box”* (student, end-of-course feedback form 2). During the course, one student realized that nurturing creativity had been a turning point in her caring journey, helping her to rethink rather than ruminate on the challenges she and her relative faced, and envisage new possibilities. After the course, they reported feeling *“more able to cope with life’s problems”* (student, end-of-course feedback form 5), inspired to find creative ways to tackle the difficulties of caring.

##### Adult education teaching styles can foster creative thinking

**Figure 10:**
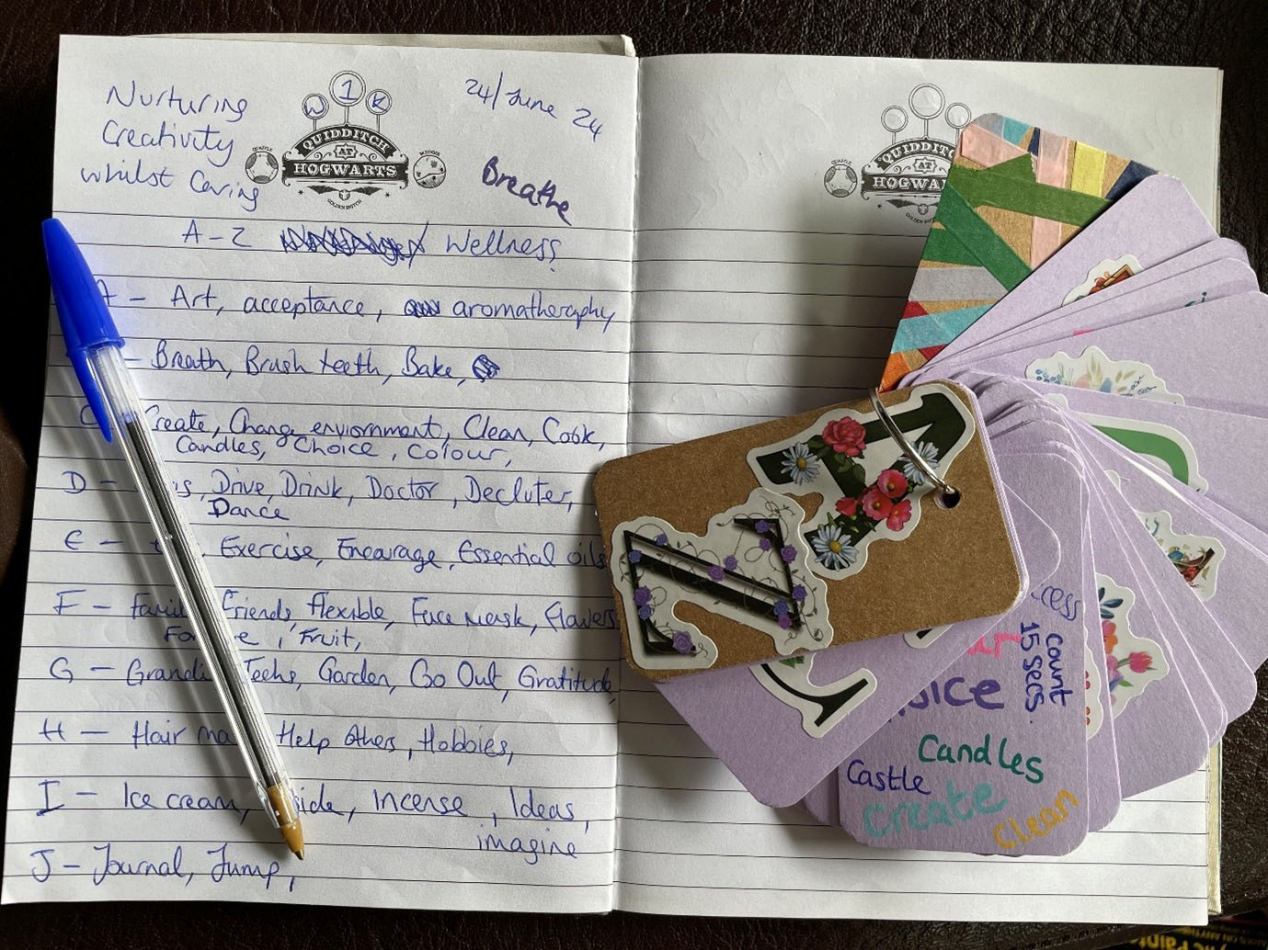
Louisa’s Wellness A-Z keyring.

Initially, some students found the ambiguity of instructions compared to other courses difficult. We reflected as tutors that we had not wanted to constrain how students expressed their creativity, so used very open prompts and questions in discussions. Some students found this confusing and preferred tasks with more structure. Meanwhile, others were surprised by the freedom and individual creativity they found when approaching this uncertainty with an open mind. *“Some more clarity about tasks being given to do e.g. the A-Z guide. However, I’m happy to see how this will evolve”* (student, session one feedback form 3). As tutors, we were amazed at how the students took the concepts that were introduced and built on them in unexpected, unique ways. For example, one student made their Wellness A-Z into a keyring (see **Figure 10**).

*Nurturing Creativity whilst Caring* put a strong emphasis on experiential learning, and we also sent additional resource packs to enable practical activities at home (as it was an online creative course). The feedback about this different way of learning was overwhelmingly positive, facilitating embedded and ongoing learning. *“It helps me sorting things and organizing my thoughts and ideas - adding colors to it and writing it down helps me process things and remember things … by making it myself its imprinted on my mind, so even if it’s not to hand I am able to think of some words that means something to me about looking after myself,”* (student quote, final session fieldnotes). This practice-based way of learning was appreciated as making real change to the family carers’ everyday lives. Asking students to reflect on their experiences after each activity further nurtured this mindset of self-discovery and exploration.

## Discussion

To explore potential ways this Recovery College course, *Nurturing Creativity whilst Caring*, could help to encourage recovery and wellbeing for family carers, we will now discuss our findings in relation to existing literature.

Since this project, another PAR study was published in Canada (34), which also developed a carer-specific Recovery College course aiming to promote their personal recovery, called We-Care-Well. Their shared methods and similar findings strengthen our argument that coproduction in Recovery College settings has great potential to further support the empowerment and wellbeing of family carers. Unlike We-Care-Well, our course specifically focused on creative ways to enable self-care. Research has found that becoming practiced at self-care is an important step in many family carers’ recovery journeys (55). Self-care for our family carers was more complex than just performing activities to maintain wellbeing (71). This resonated with the multi-dimensional framework for self-care whilst caring (originally developed for social workers) (72), which proposes numerous necessary components. We suggest our course, which encouraged creativity through coproduced recovery education, helped to facilitate some of these enablers.

One proposed requirement for self-care whilst caring is the ability to “negotiate the demands that arise from the intersection of individual and environment,” p99 (72). Early theorists such as Maslow conceptualize creativity as enabling individuals to move beyond their environment rather than simply coping with it (73), and we hoped that fostering creative ways of perceiving things may help family carers to see new ways to manage their competing demands (20). In our study some students discovered that engaging in creative activities together with the person they supported was a form of self-care whilst also caring. Indeed, previous research reported family carers found these shared creative experiences nourishing and motivating, whilst also contributing to new understandings of their caring role (74). Seeing themselves as ‘journeying alongside’ the person they support is described as important in family carers’ recoveries (55). Joint creative practices are suggested to facilitate ‘mutual recovery’ for healthcare workers, family and individuals with mental ill-health (75).

Another proposed psychological condition for self-care is “the capacity to maintain a positive and compassionate view of the self,” p99 (72). Family carers in our course discussed how to make time for themselves, they first had to let go of the guilt around what a carer should be doing and to accept their limitations. Following the course, they reported feeling kinder towards themselves. Perhaps the creative activities we incorporated into our course helped to foster self-compassion amongst the family carers (63). We also incorporated preparative mindfulness exercises to encourage an ‘openness to experience’ helpful for creativity (76, 77), but developing this non-judgemental stance may also contribute to family carers’ self-compassion (78). Previous interventions for caregivers of dementia found that creative activities allowed family carers to explore and express their identity and find a sense of mastery (57). Indeed, students described how doing creative activities reminded them of who they were before they began caring. Rediscovering/developing an identity separate from being a carer is an important part of recovery (32, 34). However, maintaining well-being and being able to draw on one’s capabilities and overcome self-criticism are suggested as prerequisites for creativity (79). This therefore supports the design of our course, which addressed both these elements simultaneously.

Engaging in creative activities together with other caregivers is shown to foster emotional and social connectedness (57), and may help develop a sense of shared identity, important for empowerment (48). Sharing and learning from peers is highlighted as one of the key mechanisms of change for Recovery Colleges (42), and may be particularly important for family carers facing social isolation (80). We found that students appreciated meeting others with similar experiences, which helped them to feel validated and encouraged to be kinder to themselves (81). They also found the space to problem-solve and share strategies for balancing their needs with those of the person they support helpful (34). Our findings support arguments that individualistic notions of self-management are inadequate without the support of others (82). In this way, we suggest that for family carers, self-care is rooted in original notions of community self-care (83), which can be facilitated in Recovery College contexts.

Delivering a course exclusively for family carers is somewhat misaligned with the Recovery College philosophy of shared learning (40), which has many benefits. For example, learning from the lived experiences of service users in the class can spark hope (84) (45). A mixed group of students might also allow healthcare professionals to become more aware of family carer needs, to improve collaborations (85). There are some reports in previous Recovery College research of family carers being apprehensive of the shared course format (86). But after our course, students said they intended to attend more courses from the wider curriculum. Eventually, this could increase family carer presence in all Recovery College courses, which would likely reduce their feelings of being a minority who don’t quite belong (44). However, further research into these longer-term effects is needed.

Overall, feedback on the online and short format of our course was mixed, demonstrating the diversity amongst those with caring responsibilities. The practical activities we incorporated may have helped to keep sessions enjoyable and engaging despite being virtual (87). Unfortunately, sending creative packs, providing additional reminders, and support with technical difficulties may not be sustainable for Recovery Colleges to implement in future. However, the homework tasks and creating physical reminders of their learning helped students continue and transfer their learning. We-Care-Well, which shared our format, also included a strong emphasis on practical takeaway tasks, such as creating Wellness Toolkits (34). They too combined these with opportunities for self-reflection throughout the course, which facilitated this transformative learning (34). Corresponding to the recovery educational pedagogy of ‘learning in praxis’ (88), our students continued to apply new ways of seeing the world more widely in their lives. Strategies to foster their creative thinking (89) appeared to overlap with those of adult and recovery education (38), making Recovery Colleges ideal conduits for teaching creativity to promote family carers’ self-care and wellbeing.

### Strengths and limitations

Whilst this evaluation focused on the perspective of the students, a strength of the chosen PAR approach was that everyone found it a meaningful and empowering process. The course tutors developed a strong friendship during the codesign, learning alongside the students and nurturing their own creativity and self-care. The Family Carers’ Advisory Group appreciated the space created for exploring the emotions and challenges of living with family and friend mental ill-health and found special significance in the various positive outcomes they had helped to facilitate for similar others by creating the course.

Due to the ongoing and embodied nature of PAR, it is difficult to disentangle the research teams’ influence on subsequent findings. In this instance, the academic researcher was involved in the codesign of the project and course content, the facilitation of the course, contributing their reflections to the data, and the analysis. This allowed us to capture ‘liveness’ of the course, helpful for researching creative experience (90), but because we did not record the discussions, some of the meaning of students’ language may have been lost or misinterpreted. We acknowledge there may be alternative ways to create a safe, empowering space whilst also recording, which could form a complementary approach to evaluating courses in future. By collecting large amounts of data, it was also difficult to ensure all of this fed into the interpretive process, although using framework analysis helped us to attempt this. We hope that the credibility of our findings was improved by CDA, triangulating multiple data sources, and checking themes with students (who reported findings were an accurate representation).

Designing any course is a delicate balance between tailoring to a specific group and making content broad and inclusive. We did not explore how individual differences affected student experiences of the course, for example, their previous arts participation (91), or relationship to the person they cared for (12). An important limitation of our study was that our sample was entirely white, female, and not in work, which likely skewed our findings and limited cultural transferability. For example, the psychological conditions for self-care and creativity featured heavily in the course discussions, as opposed to the service level or socioeconomic barriers common for many family carers (92). The peer tutor also reflected that despite having her own experiences of caregiving, she was still surprised by different student feedback, recognizing the difficulty of representing family carers (49). However, the multiple people involved in coproducing the project brought diverse expertise and experience, contributing critical perspectives.

We did not follow up on whether the benefits described after the course were long-lasting, which would have supported claims that our teaching strategies encouraged continued development in family carers’ recovery journeys. However, one student corresponded with the academic researcher nine months after the course, reporting that she had continued to make time for herself and think creatively about different things she could try. Our study was exploratory in nature, and we did not aspire to demonstrate outcomes of an intervention, so we did not use any pre- or post-measures. Rather, we used a rigorous process to reflect on experiences of a single course, hoping to draw insights into how to support family carers’ wellbeing through educational Recovery College courses in future. Replicating the course may provide support for our conclusions, but each iteration is likely to vary depending on the unique synergy between tutors and students.

### Implications for future research and practice

For family carers, we propose a conceptualization of self-care as a way of perceiving and relating to the self, the person they care for, and their wider community. Acknowledging complexity may be an important premise for future interventions that aim to increase family carer engagement in wellbeing behaviors, including the seeking and attendance of services and wider support. Some PAR studies incorporate a dissemination event, where those involved in the project share their learning with wider audiences, and this is shown to be empowering and increase impact (59). Adding this to the design of our course, for example by creating a booklet to share or an exhibition with images of the students’ self-care and creativity, could deepen understanding amongst healthcare professionals, despite the carer-only format of the course.

We highlight some potential active ingredients to nurturing self-care amongst family carers, such as mindfulness, creativity and peer learning spaces, but future studies could more rigorously test these. Combined with existing models of creativity and self-care developed for family carers of people with dementia (93) and general mechanisms of action for Recovery College students (42), our findings provide direction for family carers and academics to cocreate a theory of change for how coproduced creative learning in Recovery Colleges can promote wellbeing for mental health family carers.

Substantial time and resources were invested in coproducing our course, which is not uncommon, as a survey of Recovery Colleges across England found the mean cost of designing a course was £8101 (37). It would also be useful to investigate whether the course can be upscaled, perhaps opening this up to family carers in other parts of the country. By disseminating the rationale and outcomes from our course, we hope it can then be delivered multiple times and shared with other Recovery Colleges. Moreover, we hope our exploration of the students’ experiences of our teaching methods can contribute to the currently lacking literature on the pedagogy of Recovery Colleges (94). Further research into the practical implementation of educational theories in these settings is required. For example, we received mixed feedback from students about our balance between giving specific instructions and structure with providing freedom for individuality and unpredictability (95), which may have limited some of the students’ growth in self-confidence. This knowledge would help ensure teaching strategies suited all students, as well as help Recovery Colleges translate their courses to online delivery (an area our students felt could be improved). Development of evidence-based toolkits for course design and delivery would also help instill quality and cost-effectiveness across provision.

## Conclusions

This study provides a detailed description for how PAR can help to design, deliver and evaluate an educational course promoting recovery amongst family carers within a Recovery College. We specifically chose to focus on transformational and experiential ways of nurturing creativity and self-care. Students described how experimenting with creative activities and reflecting together during the course helped them to develop a more accepting and curious approach, which they went on to apply to themselves, their caring role, and their daily lives. We hope that the mutually reinforcing nature of self-care, self-compassion, mindfulness and creativity will lead to longer-term benefits for the family carers. However, more research is needed to measure these outcomes and establish the active ingredients, so that this course can be replicated in other Recovery College contexts to reach more family carers.

## Data Availability

The raw data supporting the conclusions of this article will be made available by the authors, without undue reservation.
